# Bridging the Gap in Health Personnel and Elderly Communication Training: What Can We Learn From Speech Codes Theory

**DOI:** 10.7759/cureus.21659

**Published:** 2022-01-27

**Authors:** Beheshta Momand, Brenda Barth, Winnie Sun, Adam Dubrowski

**Affiliations:** 1 Health Sciences, Ontario Tech University, Oshawa, CAN

**Keywords:** education, elderly individuals, healthcare simulation, communication, speech codes theory

## Abstract

Effective communication in healthcare settings allows for the expression of complex or technical terms in a manner that each patient can understand. Communication is also linked to increased trust, patient and family satisfaction, and mutual agreement between patients and healthcare personnel. As a result of aging, the elderly (age 65 and older) may develop physical, cognitive, and social changes that may lead to barriers when interacting with healthcare personnel. As a result of these age-related changes, the elderly ability to receive, retain, and convey information may be affected. Therefore, it is essential that healthcare personnel use appropriate language when communicating with this population. Studies have suggested that simulation can be an effective means to train healthcare personnel to develop context-appropriate communication skills for this specific population. This editorial will explore how the Speech Codes Theory (SCT) can structure simulation encounters to enhance healthcare personnel's proficiency in conversing and connecting with this patient population.

## Editorial

Background

It is well accepted that effective communication between two individuals who do not share a common language is difficult or often impossible. It is also well received that strategies that work to improve the effectiveness of communication between two individuals who speak the same language, such as speaking slower or louder, will not work when a common language is not shared. In this editorial, we argue that barriers to effective communication between health personnel and elderly patients may be very similar to communication barriers between two individuals who speak different languages. Therefore, to improve the effectiveness of the communication in this context, unique communication strategies and underpinning educational approaches need to be introduced. One theory that may help with the construction of educational programs to improve health personnel-elderly patient communication is Speech Codes Theory (SCT) [1]. SCT is a communication framework that explores an individual's communication based on societal, cultural, gender, occupational, or other factors [1].

Communication dramatically impacts a patient's perception regarding the quality of care they receive. Research supports the numerous benefits of effective communication and its influence on patients' health outcomes. Patient-centered care emphasizes the essential features of therapeutic communication, which allows healthcare personnel to share information, thoughts, and concerns with patients, patient families, and other healthcare personnel [1]. In relation to health personnel-patient relationships, effective communication protects patients from potential harm by receiving, retaining, conveying, and understanding information essential to promoting patient care. According to the Health Canada Survey, 25% of Canadian healthcare facilities, colleges, and personnel associations underscored communication as the leading cause of documentation errors and medical errors, significantly impacting patient safety [2].

It has been suggested that communication between health personnel and the elderly can be challenging due to a lack of training and education within the health sciences curriculum. Without the proper education in communication, it becomes difficult for health personnel who interact with elderly patients to know when to ask probing questions, how to address their needs, and how to deliver sensitive information. One educational approach that may be well suited for developing communication skills is experiential learning, specifically simulation-based education (SBE). This paper illuminates a novel way to use simulation to develop competency in health personnel-patient communication through the lens of SCT.

Current gap

Simulation, defined as a replication of a task or an event for the purpose of competency development and/or assessment, is one way that we can improve communication between health personnel and elderly patients [3]. It allows educators to replace real-life experiences encountered in a clinical setting with simulated ones in order to address possible patient safety concerns when untrained health personnel develops skills on real patients. SBE is accepted as an effective way to develop a range of technical and non-technical skills, such as communication strategies while protecting both the learners and patients from unnecessary risks of harm.

However, current research in the area of utilizing SBE to train health personnel's - elderly patient communication is either focused on dealing with the physiological and cognitive aspects of aging, adopting a multidisciplinary approach, as well as looking explicitly at assessment questions, or planning and structuring the communication. For example, Foronda et al. [3] developed a virtual simulation to improve communication skills in Bachelor of Nursing (BSN) students. The study used the Identify-Situation-Background-Assessment-Recommendation (ISBAR) communication technology to improve nursing students' communication. This issue with using the ISBAR technique is that it focuses on ways health personnel can plan and structure communication rather than how they can communicate with elderly patients. The how component of communication is crucial because it considers the words used when to use them, and which words are more appropriate to use when conversing with elderly patients. Therefore, as comprehensive as this tool may be, it does not adequately educate health personnel on the effective skills needed to establish therapeutic communication with older adults.

Proposed solution

Currently, there are no known theoretical frameworks for SBE for healthcare providers who focus on the social and language aspect of communicating with the elderly. One theory that can be used to structure SBE to educate and improve health personnel's communication skills is the SCT [4]. This theory implies that each culture has a distinct way of speaking, and the words spoken to express concepts and beliefs are referred to as speech codes [4]. These codes are how interpersonal communication is produced, translated, processed, and understood. The SCT suggests that individuals encounter multiple speech codes throughout their lifetime and that these speech codes are related to the people and relationships of that culture. Through the lenses of SCT, culture refers to the “socially constructed and historically transmitted pattern of symbols, meanings, premises and rules” [4].

Although SCT has not been used as part of healthcare personnel's elderly communication training, it has been used as a lens to conceptualize how health personnel communicates with each other. In a study that examined communication between urban and rural physicians during consultations [5], it was found that both groups of physicians perceived that they experienced challenges in understanding each other's contexts (e.g., depth of familiarity with a patient). The language and jargon used (i.e., speech codes) were the main reasons for suboptimal communication patterns that often led to suboptimal patient outcomes. To the best of our knowledge to date, SCT has not been used as a theoretical underpinning in SBE, especially in the context of health personnel-elderly patient communications. Therefore, we suggest that SCT has the potential to be embedded within SBE to address and accommodate the cultural needs of this patient population. SCT recommends that individuals alter their speech codes depending on the individual or group they are conversing with. Using speech codes that are familiar to a community can allow for an understanding, connection, empathy, and acknowledgment. One possible way to operationalize the use of SCT in SBE is through the inclusion of the related concepts in virtual online simulation and a debriefing session to reflect on the experiential learning. Specifically, the virtual online simulation may incorporate the types of speech codes that the elderly often use. Similarly, the inclusion of SCT in the debriefing process may allow learners to gain an understanding of their thought processes, promote learning outcomes, and be able to identify areas of improvement in communication techniques.

Conclusion

Effective communication is foundational to high-quality healthcare. It is critical to meet patient needs and provide safe, patient-centered care. It allows for the exchange of information, thoughts, and concerns by means of verbal and non-verbal means. Poor communication can lead to many adverse health outcomes, such as patient injuries, medication errors, delays in essential tests and treatments. When accessing and utilizing health services, the elderly are vulnerable populations due to the challenges associated with the aging process. Ineffective communication can result in elderly patients feeling inadequate, disempowered, and helpless. Studies have indicated that simulations are an effective way to train health personnel in the development of communication skills. We propose that by embedding SCT within SBE, we have the potential to transform the educational curriculum related to communication with older adults in diverse healthcare settings.
